# Supplementary material for: Sustainable energy management using the Internet of Things (IoT)
Source: PLoS One. 2023 Jun 20;18(6):e0283754. doi: 10.1371/journal.pone.0283754 (PMC10281580; doi:10.1371/journal.pone.0283754)
Supplement: Attachment — Submitted filename: Reply to Referres Comments.docx [file pone.0283754.s001.docx]

The Editor in Chief,

PLOS ONE	Date: 21^st^ February 2023.

**Reference: revised manuscript submission for paper id no** PONE-D-23-00228

Dear Editor,

Thank you for your positive response and for allowing a resubmission of our manuscript, with an opportunity to address the reviewers’ comments. Following your email dated 7^th^ February 2023 regarding the manuscript " Sustainable Energy Management Using the Internet of Things (IoT)." with Code Number PONE-D-23-00228, we thank you for arranging a peer review of the article. We would also like to thank you for your encouraging and supportive comments regarding our paper.

We found the reviewers’ comments very helpful and constructive. In the light of these comments, the manuscript has been fully revised.

Please find attached the detailed response to the reviewers’ comments, highlighting the changes performed to the manuscript. We are confident that the new manuscript version has a more fluent scientific discourse. Please note that most of the corrections made are per the evaluation.

For clarity, the convention is tabulated below:

| Category | Description |
| --- | --- |
| Reviewers’ comments | **bold text encompassed in “double quotes”** |
| Authors’ response | blue font |
| Text added/edited (English)  Text added/edited (Technical) | In the manuscript, some of the English and technical changes or small revisions of sentences etc. are indicated through track changes”. |

Best regards,

Shahbaz Khan (Corresponding Autor and on behalf of all co-authors)

Department of Mechatronics Engineering

University of Engineering & Technology, Peshawar Pakistan

**Response to Reviewers' Comments**

We would like to extend our warm regards to the reviewers for sharing their expert opinions and useful comments.

This is indeed a great pleasure for us to see that all the reviewers are overall positive about the manuscript. The comments suggested by the reviewers have helped us to further improve the paper. Here are the point-wise details of the revision. The response has been highlighted in **blue** font.

**Editor Comments**

**1. “Please ensure that your manuscript meets PLOS ONE's style requirements, including those for file naming.”**

**The authors thank the editor for the positive remarks and constructive feedback. The manuscript has been updated according to PLOS ONE’s style.**

**2. “Please note that PLOS ONE has specific guidelines on code sharing for submissions in which author-generated code underpins the findings in the manuscript. In these cases, all author-generated code must be made available without restrictions upon publication of the work.”**

**The authors thank the editor for the positive remarks and constructive feedback. The code has been shared in github with the following link.**

[**https://github.com/MIANHAZRATSHAH/Sustainable-Energy-Management-Using-IOT/blob/48e3c37a980b47e405c240bdc89ae9ccb5bc7291/Sustainable_Energy_Management_using_IOT.ino**](https://github.com/MIANHAZRATSHAH/Sustainable-Energy-Management-Using-IOT/blob/48e3c37a980b47e405c240bdc89ae9ccb5bc7291/Sustainable_Energy_Management_using_IOT.ino)

**3. “ Thank you for stating the following financial disclosure:**

**"The funders had no role in study design, data collection and analysis, decision to publish, or preparation of the manuscript."**

**At this time, please address the following queries:**

**a) Please clarify the sources of funding (financial or material support) for your study. List the grants or organizations that supported your study, including funding received from your institution.**

**b) State what role the funders took in the study. If the funders had no role in your study, please state: “The funders had no role in study design, data collection and analysis, decision to publish, or preparation of the manuscript.”**

**c) If any authors received a salary from any of your funders, please state which authors and which funders.**

**d) If you did not receive any funding for this study, please state: “The authors received no specific funding for this work.”**

**Please include your amended statements within your cover letter; we will change the online submission form on your behalf.”**

**The authors thank the editor for the positive remarks and constructive feedback. The authors have received no specific funding for this work. This statement is also written in the updated cover letter.**

**4. “In your Data Availability statement, you have not specified where the minimal data set underlying the results described in your manuscript can be found. PLOS defines a study's minimal data set as the underlying data used to reach the conclusions drawn in the manuscript and any additional data required to replicate the reported study findings in their entirety. All PLOS journals require that the minimal data set be made fully available. For more information about our data policy, please see http://journals.plos.org/plosone/s/data-availability.”**

**The authors thank the editor for the positive remarks and constructive feedback. All the relevant data has been shared in the manuscript, and no other data in this research needs to be made available.**

**5. “We note that Figure 4 and 11 to 18 in your submission contain copyrighted images. All PLOS content is published under the Creative Commons Attribution License (CC BY 4.0), which means that the manuscript, images, and Supporting Information files will be freely available online, and any third party is permitted to access, download, copy, distribute, and use these materials in any way, even commercially, with proper attribution. For more information, see our copyright guidelines: http://journals.plos.org/plosone/s/licenses-and-copyright.”**

**The authors thank the editor for the positive remarks and constructive feedback. All the figures have been changed in the revised manuscript. The last two figures that are 19 and 20 are basically taken during the testing of the prototype.**

**6. “Please ensure that you refer to Figure 1, 5, 6, 15 to 18 in your text as, if accepted, production will need this reference to link the reader to the figure.”**

**The authors thank the editor for the positive remarks and constructive feedback. All the figures have been referred to and linked in the revised manuscript.**

**7. “We note you have included a table to which you do not refer in the text of your manuscript. Please ensure that you refer to Table 1, 3 to 5 in your text; if accepted, production will need this reference to link the reader to the Table.”**

**The authors thank the editor for the positive remarks and constructive feedback. All the tables have been referred and linked to in the revised manuscript.**

**Reviewer 1:**

**1. “This research focuses on energy management, making the distribution authority more powerful, digitalization and protection of expensive components in electrical power systems. The proposed methodology uses current and voltage sensors to remotely monitor the amount of power being supplied to the consumer continuously, along with a microcontroller responsible for activating the relay in case of over-consumption and the Global System for Mobile (GSM) network to warn the consumer and inform the authority. In general, there is no scientific contribution in this paper. The authors are encouraged to clarify the constraints and the cost of practical implementation for each consumer”.**

**The authors would like to thank the reviewer for the positive remarks and constructive feedback. As suggested by the reviewer, the authors have revised their manuscript significantly. As suggested, the revised manuscript's main contribution has been highlighted through track changes. The revised text has also been reproduced below verbatim.**

**“The main contribution of this proposed research is to protect the expensive elements like a transformer from consumer’s side, while considering sustainability and not using chemicals, gases, or oil on transformer’s side.”**

**The authors have also compared the developed system with the readily available smart meters in the market, as shown in table 7 of the revised manuscript. The main constraint in adopting the concept is the conventional mindset of the consumer. However, the developed system is significantly cheaper than the existing smart meters.**

**Reviewer 2:**

**“A real time working model as mentioned can be added with some snapshots of the device working with GSM. Cost specification to be clear on a broader end of usage. Software utilized specification to be added. Critical review of previous available works/ relative works can be added to increase the application feature”.**

**The authors would like to thank the reviewer for their appreciation. The reviewer's comments have helped us further improve the paper. The authors have compared the developed system with the existing smart meters in table 7 of the revised manuscript. The cost specification of the developed system and its comparison with the readily available smart meter is also highlighted in the revised manuscript through track changes.**

**The specification of the software utilized has also been added in the revised manuscript and highlighted through track changes.**

**The critical review of the literature has been summarized in table 3 of the revised manuscript and highlighted through track changes.**

**--------------------- End of Document-----------------------**
